# Extracellular vesicles from genetically unstable, oncogene-driven cancer cells trigger micronuclei formation in endothelial cells

**DOI:** 10.1038/s41598-020-65640-7

**Published:** 2020-05-22

**Authors:** Shilpa Chennakrishnaiah, Thupten Tsering, Caroline Gregory, Nadim Tawil, Cristiana Spinelli, Laura Montermini, Nicolaos Karatzas, Saro Aprikian, Dongsic Choi, Ludger Klewes, Sabine Mai, Janusz Rak

**Affiliations:** 1 Research Institute of the McGill University Health Centre, McGill University, Montreal, QC H4A 3J1 Canada; 2 Department of Cell Biology, Research Institute of Oncology and Hematology, Cancer Care Manitoba, University of Manitoba, Winnipeg, Canada

**Keywords:** Cancer, Cell biology, Genetics

## Abstract

Oncogenic transformation impacts cancer cell interactions with their stroma, including through formation of abnormal blood vessels. This influence is often attributed to angiogenic growth factors, either soluble, or associated with tumor cell-derived extracellular vesicles (EVs). Here we examine some of the cancer-specific components of EV-mediated tumor-vascular interactions, including the impact of genetic driver mutations and genetic instability. Cancer cells expressing mutant *HRAS* oncogene exhibit aberrations of chromatin architecture, aneuploidy, cytoplasmic chromatin deposition and formation of micronuclei with a non-random chromosome content. EVs released from such *HRAS*-driven cells carry genomic DNA, including oncogenic sequences, and transfer this material to endothelial cells while inducing abnormal formation of micronuclei, along with cell migration and proliferation. Micronuclei were also triggered following treatment with EVs derived from glioma cells (and stem cells) expressing EGFRvIII oncogene, and in both endothelial cells and astrocytes. EVs from HRAS and EGFRvIII-driven cancer cells carry 19 common proteins while EVs from indolent control cells exhibit more divergent proteomes. Immortalized endothelial cell lines with disrupted TP53 pathway were refractory to EV-mediated micronuclei induction. We suggest that oncogenic transformation and intercellular trafficking of cancer-derived EVs may contribute to pathological vascular responses in cancer due to intercellular transmission of genomic instability.

## Introduction

Formation of the vascular tumor stroma defines crucial events in cancer progression, including invasive growth, metastasis, interactions with the immune system, paraneoplastic states (e.g, thrombosis) and responses to therapy^1^. A wide range of endothelial cell responses associated with the malignant process are exemplified by (and often reduced to) the angiogenic vascular growth program involving a network of canonical effectors including vascular endothelial growth factor (VEGF-A) and its receptors, as well as several other pathways essential for physiological vascular growth^2,3^. While targeting this circuitry was postulated to give rise to promising anticancer therapies^4^ and led to derivation of effective VEGF pathway antagonists^1^, several vascular and metastatic tumors do not respond to this form of treatment^5^ raising questions as to alternative forms of vascular supply^6^ and alternative, cancer-specific vascular regulators.

Oncogenic transformation profoundly changes the ability of affected cells to interact with their microenvironment, including blood vessels^7^, blood components^8^, stroma^9^ and the immune system^10^. While this is often attributed to deregulation of the respective soluble mediators, such as VEGF or interleukins^7,11^, activation of oncogenic RAS or EGFR also impacts the particulate (insoluble) secretome of cancer cells including the composition of extracellular vesicles (EVs) and particles (EPs)^12^. EVs are heterogenous, mostly spherical, cellular fragments ranging from ~50 nm to >2 µm in size and surrounded by the plasma membrane^13^. Depending on their subset EVs are either shed from the cell surface as larger microvesicles (MVs), or expelled into the extracellular space from within the multivesicular endosome (MVB) as small EVs known as exosomes^14^ with considerable differences in molecular cargo and biological properties^15^. EVs represent a unique mechanism of molecular expulsion, whereby soluble and insoluble bioactive macromolecules (proteins, RNA, DNA) can be removed from cells into the microenvironment, and thereby lose their intracellular effects^16^. Once released EVs enter interstitial fluid space, biofluids and blood, where they are transmitted over large distances and interact with different cellular populations, including endothelial cells^17,18^.

Several lines of evidence suggest that cancer EVs contribute to oncogene-driven vascular pathology and angiogenesis. This includes transfer of the pro-angiogenic phenotype between cancer cell subpopulations through the exchange of EVs carrying oncogenic EGFR (oncosomes)^19^. EGFR-carrying cancer EVs can also interact with endothelial cells directly and reprogram their signaling circuitry and growth factor responses^20^. These effects are of interest as they may suggest the engraftment of cancer-specific molecular machinery into endothelial cells and non-canonical regulation of tumor angiogenesis.

Notably, protracted oncogenic signaling exerts a profound and irreversible influence on the genome and epigenome of cancer cells^21,22^, effects with presently unknown consequences for the vascular^8^ and angiogenic activity of cancer EVs. Here we show that cancer cells harboring oncogenic HRAS exhibit considerable genetic instability, as indicated by the formation of micronuclei and release of EVs carrying genomic DNA. These EVs can be taken up by primary endothelial cells and stimulate their migration coupled with formation of micronuclei and induction of stress responses. Micronuclei in endothelial cells were also induced by EVs of other types of cancer cells (mesenchymal human glioma stem cells, EGFRvIII driven glioblastoma) but not by EVs from isogenic normal or more indolent cells. Our results suggest that the angiogenic responses of vascular stroma to oncogenic EVs are distinct and may contain anomalies such as induction of genetic instability.

## Results

### Oncogenic RAS triggers aberrations in the chromatin architecture

**Figure 1:**
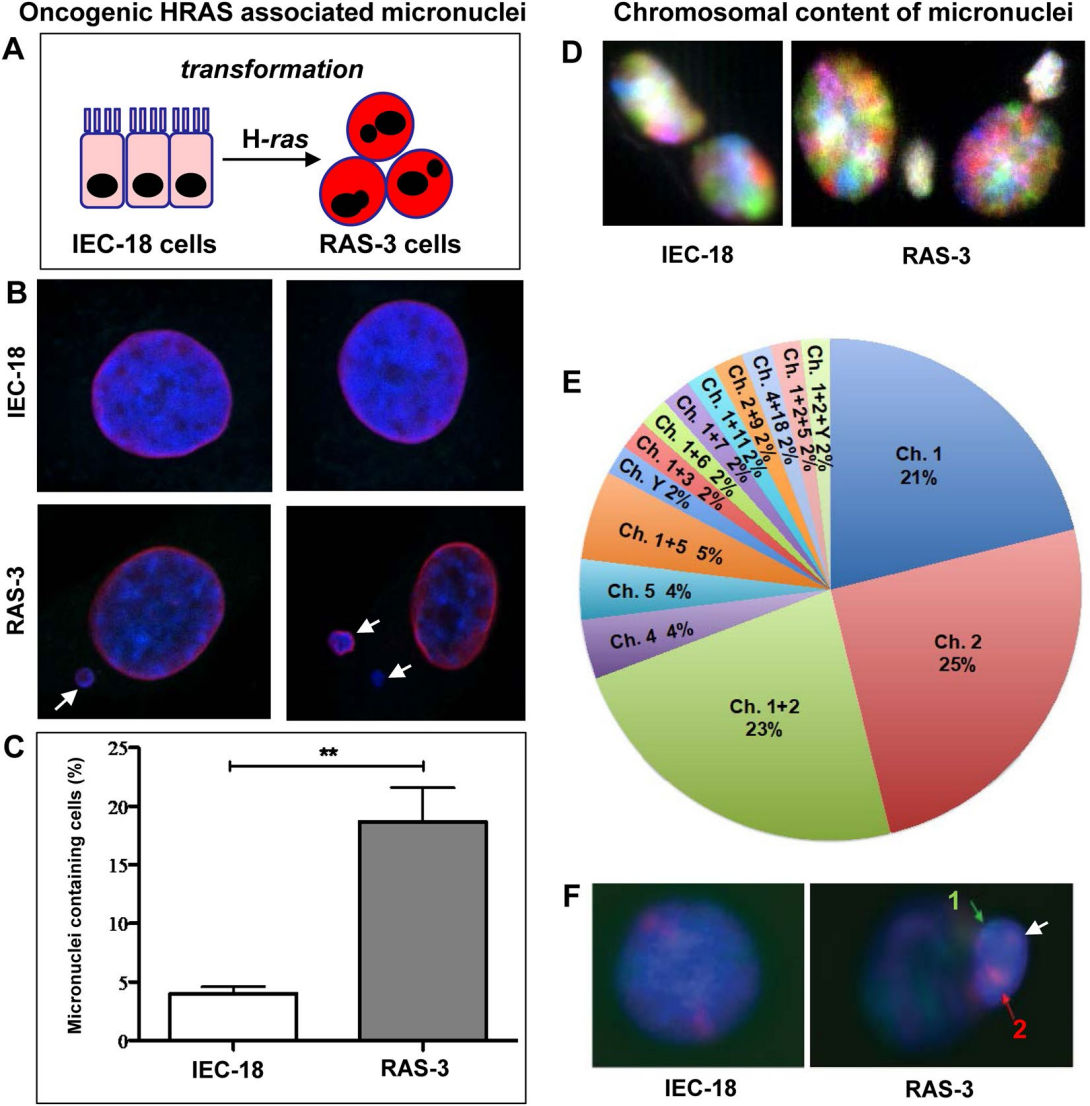
HRAS transformation triggers formation of micronuclei with chromosome enrichment. (**A)** Derivation of RAS-3 cells from HRAS-transformed IEC-18 epithelial cell line. (**B)** Micronuclei formation by RAS-3 cells (DAPI – blue; lamin B1 staining – red). (**C)** Quantification of micronuclei in IEC-18 and RAS-3 cells ** p < 0.01. (**D)** SKY staining of IEC-18 and RAS-3 nuclei and micronuclei. (**E)** Contribution of chromosomes to micronuclei in RAS-3 cells. (**F)** FISH – chromosomes 1 (green) and 2 (red) in RAS-3 micronucleus.

**Figure 2:**
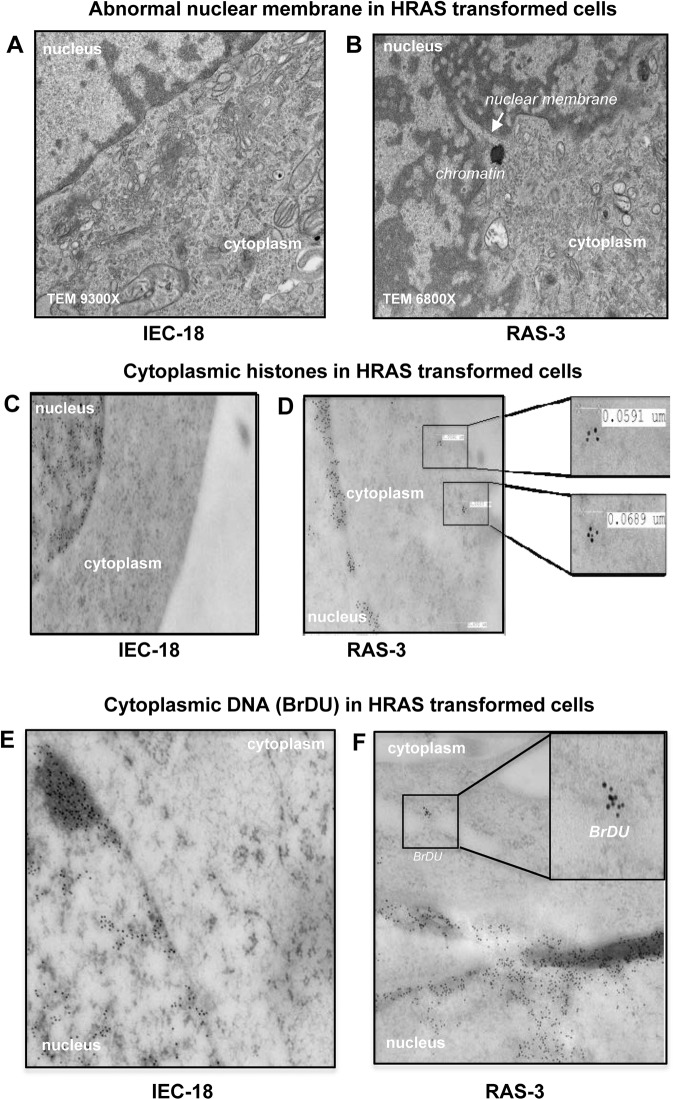
HRAS transformation leads to aberrations of nuclear membrane and accumulation of cytoplasmic chromatin in cancer cells. (**A,B)** TEM of nuclear-cytoplasmic boundary in IEC-18 and RAS-3 cells; disrupted nuclear envelope in RAS-3 cells. (**C,D)** Immunogold staining for histone; presence of cytoplasmic chromatin in RAS-3 cells (insets - high power 30,000X images of cytoplasmic chromatin deposits). (**E,F)** Immunogold - cytoplasmic staining of RAS-3 cells with anti-BrDU antibody indicates the presence of extranuclear newly synthesized DNA (insets - high power 18,500 X images of cytoplasmic BrdU positive material).

We have previously observed that transformation of non-tumorigenic rat intestinal epithelial cells (IEC-18) by expression of human *HRAS* oncogene triggers highly aggressive behavior in the resulting family of cell lines (e.g. RAS-3)^7^ (Fig. 1A) along with the increased emission of EVs containing genomic DNA^23^. We reasoned that the entry of chromatin into the extracellular space through the EV compartment of viable HRAS-transformed cells would require some alterations in the nuclear architecture (Figs. 1, 2; Supplementary Figs. S1–S4)^24^. Indeed, confocal imaging of the nuclei of IEC-18 and RAS-3 cells using staining with DAPI (DNA) and anti-Lamin-B1 antibody (nuclear envelope; Fig. 1B,C), spectral karyotyping (SKY; Fig. 1D,E; Supplementary Figs. S1, S2), fluorescent in situ hybridization (FISH; Fig. 1F) and transmission electron microscopy (TEM; Fig. 2) revealed several dramatic differences. For example, unlike their IEC-18 counterparts, HRAS-transformed RAS-3 cells exhibited high frequency of abnormal mitoses and micronuclei formation (Fig. 1B–D; Supplementary Figs. S1–S3) often with preponderance of large chromosomes. Thus chromosomes 1 (21%), 2 (25%) and a combination of chromosomes 1 and 2 (23%), account for 69% of chromosomes included in RAS-3 derived micronuclei (Fig. 1E; Supplementary Figs. S2 and S4). Interestingly, the majority of remaining 31% micronuclei also contain chromosome 1 (17%) or to some extent chromosome 2 (6%) in combination with other small chromosomes (Fig. 1E), and with a lesser contribution of chromosomes 4, 5 (at 4% each; Fig. 1E). RAS-3 cells also exhibited folds and alterations in their nuclear envelope (Fig. 2A,B) with an increased presence of histones and BrDU-positive DNA deposits in the cytoplasm, as revealed by immunogold staining with respective antibodies (Fig. 2C–F).

### Extracellular release of genomic DNA by HRAS transformed cancer cells, a possible role of autophagy

**Figure 3:**
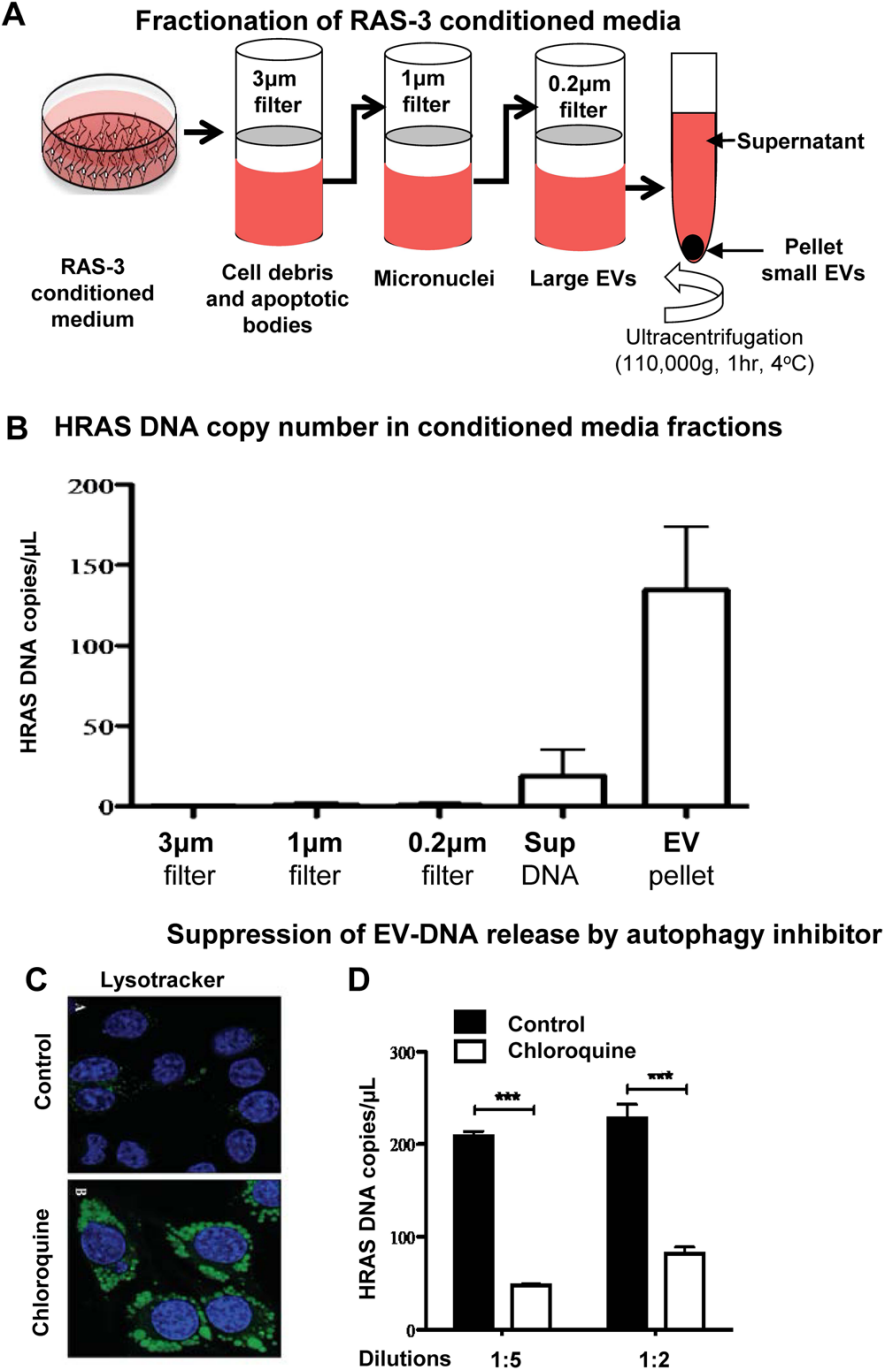
Extracellular vesicle (EV)-mediated emission of genomic DNA sequences from RAS-3 cells is sensitive to autophagy inhibitor chloroquine. (**A)** Filtration/ultracentrifugation protocol to separate putative carriers of extracellular DNA (apoptotic bodies, microvesicles, small vesicles, soluble DNA). (**B)** *HRAS* DNA copy number per microliter of conditioned media in fractions (defined in panel A). (**C)** Lysotracker staining indicates retention of the dye in lysosomes of cells treated with chloroquine (inhibition of autophagy). (**D)** Chloroquine reduces the content of *HRAS* DNA in the EV fraction of RAS-3 conditioned media; ***p < 0.001.

This pattern of large-scale genomic aberrations and cytoplasmic displacement of chromatin and micronuclei, including to regions proximal to the plasma membrane, could play a role in extracellular release of genomic DNA reported earlier for RAS-3 cells^23^. To assess whether this material originates from extrusion of micronuclei^25^, formation of DNA-containing EVs^23^ or involves release of soluble DNA, we fractionated conditioned medium of RAS-3 cells by passing it through a series of filters to capture putative DNA-containing particles including pore sizes of 3 µm (cells), 1 µm (apoptotic bodies, micronuclei), and 0.2 µm (large EVs). The flow through was separated by ultracentrifugation (110,000 g) into pellets (small EVs) and supernatant containing soluble material (Fig. 3A). The respective fractions were then tested for mutant *HRAS* copy number (ddPCR) in relation to the starting volume of the conditioned media. Interestingly, while RAS-3 cells produced ample micronuclei this material had negligible contribution to extracellular DNA, and we only sporadically observed exit of micronuclei from live cells (data not shown). We also detected minimal amounts of DNA on other filters suggesting that large EVs and apoptotic bodies (if any) played a minor role in this DNA release process. In contrast, the vast majority of extracellular DNA produced by RAS-3 cells were associated with the ultracentrifugated pellet containing small EVs (100-150 copies/µl of media), which is in line with prior reports^23^. The remaining soluble material (supernatant) contributed less than 50 copies of *HRAS* DNA per µl of culture media (Fig. 3B).

Since autophagy was postulated to play a role in small EV biogenesis^26^, formation of cytoplasmic chromatin^24^ and release of extracellular DNA from various cells^27^, we asked whether this process was involved in HRAS-driven formation of DNA-containing EVs as well. RAS-3 cells were cultured in the presence of chloroquine, which prevents acidification of the lysosomal compartment leading to retention of the LysoTracker tracer (Fig. 3C) and impaired autophagic flux^28^. Indeed, in the presence of chloroquine the amount of EV-associated DNA was visibly reduced (Fig. 3D), while the number of EVs remained undiminished (data not shown). These results suggest that small EVs (possibly associated with autophagic machinery) may act as carriers of genomic DNA between the interior of genetically unstable cancer cells and the extracellular milieu.

### Extracellular vesicles containing oncogenic DNA stimulate endothelial cell migration

**Figure 4:**
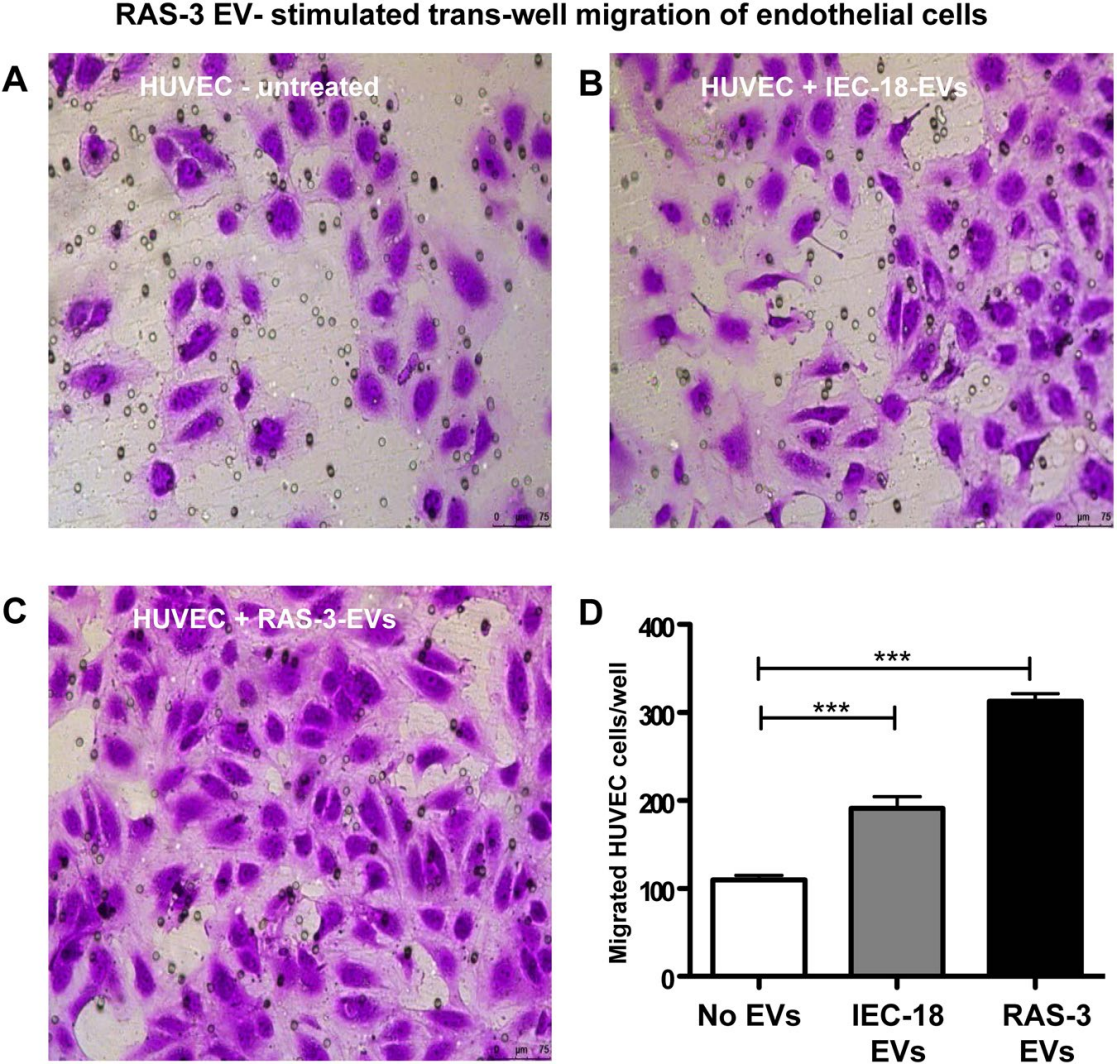
EV-induced endothelial cell migration. (**A–C)** Micrographs of migrated endothelial cells in the presence or absence of EVs. (**D)** Quantification of migrated endothelial cells; ***p < 0.0001.

Oncogenic (mutant) DNA uniquely marks EVs derived from cancer cells, and along with associated proteins and other bioactive cargo may contribute to their biological activity upon transmission to recipient cells^8,23,29^. Since endothelial cells are among the primary recipients of circulating EVs^18^ we explored their responses to the uptake of cancer EVs as a reflection of the associated angiogenic activity^19,30^ Primary endothelial cells (HUVEC) were incubated with equivalent amounts of EVs isolated from either non-transformed IEC-18 cells, or from their HRAS-transformed RAS-3 counterparts (Supplementary Fig. S5). We measured transwell migration of these cells as an approximation of one of the key aspects of blood vessel formation. While EVs from both sources stimulated endothelial cell migratory activity, RAS-3 EVs were markedly more potent (Fig. 4). This result parallels previously observed increased in endothelial cell proliferation upon exposure to EVs from RAS-3 cells^29^, human cancer cells^20^ or glioma stem cells (Supplementary Fig. S6).

### Uptake of oncogenic EVs by endothelial cells induces features reminiscent of genotoxic stress response

**Figure 5:**
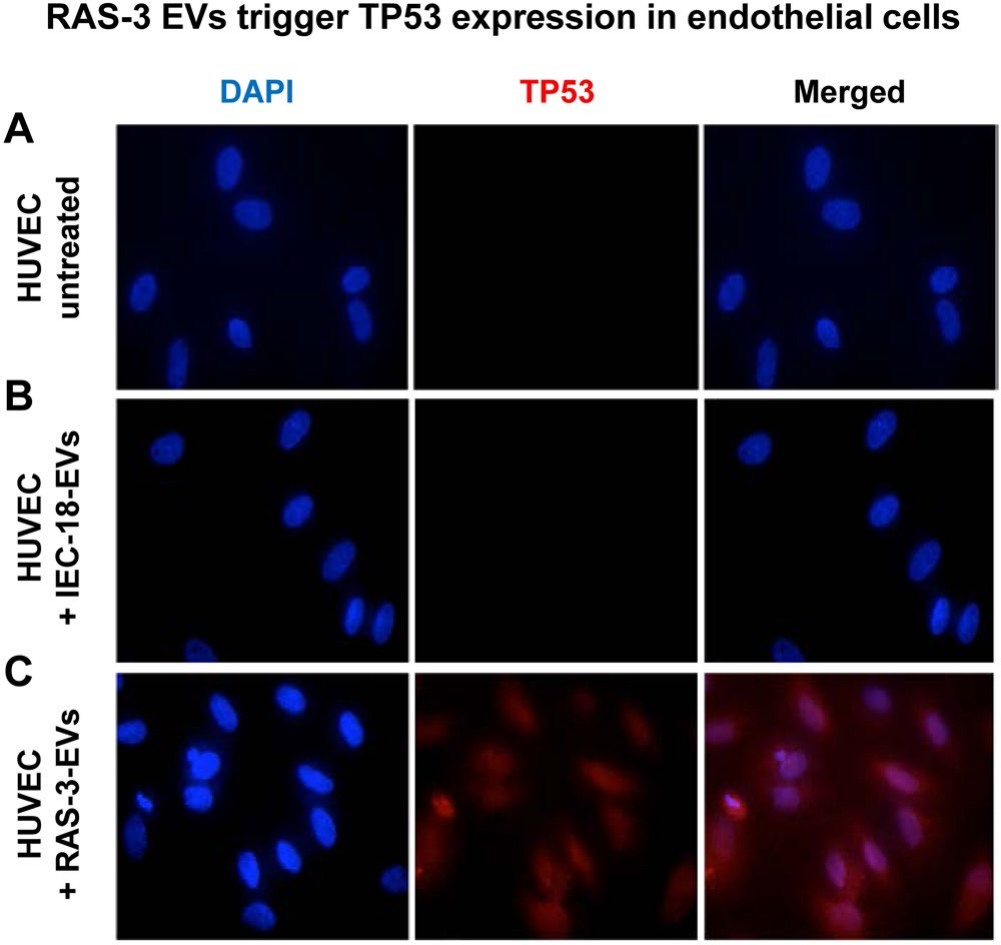
Uptake of EVs from RAS-driven cancer cells triggers TP53 expression in endothelial cells. (**A)** Untreated HUVEC. (**B)** HUVEC treated with IEC-18 EVs. **C**. HUVEC treated with RAS-3 EVs (DAPI – blue; anti-TP53 – red).

While tumor-derived EVs may transfer many proangiogenic activities to endothelial cells, their content of chromatin and oncogenic DNA signifies a cancer-specific composition with possible implications for the affected vasculature. This is relevant because of protracted (7 day long) presence of foreign DNA fragments we observed in endothelial cells exposed to RAS-3 EVs (Supplementary Fig. S5). To explore the consequences of this interaction we stained endothelial cells treated with either growth media, IEC-18 EVs or RAS-3 EVs for TP53 expression, an established marker of genotoxic stress. Interestingly, RAS-3 EVs triggered a detectable increase in TP53 signal, which was low or undetectable in control endothelial cells (Fig. 5).

### Endothelial cells exposed to oncogenic EVs exhibit overt genomic alterations

**Figure 6:**
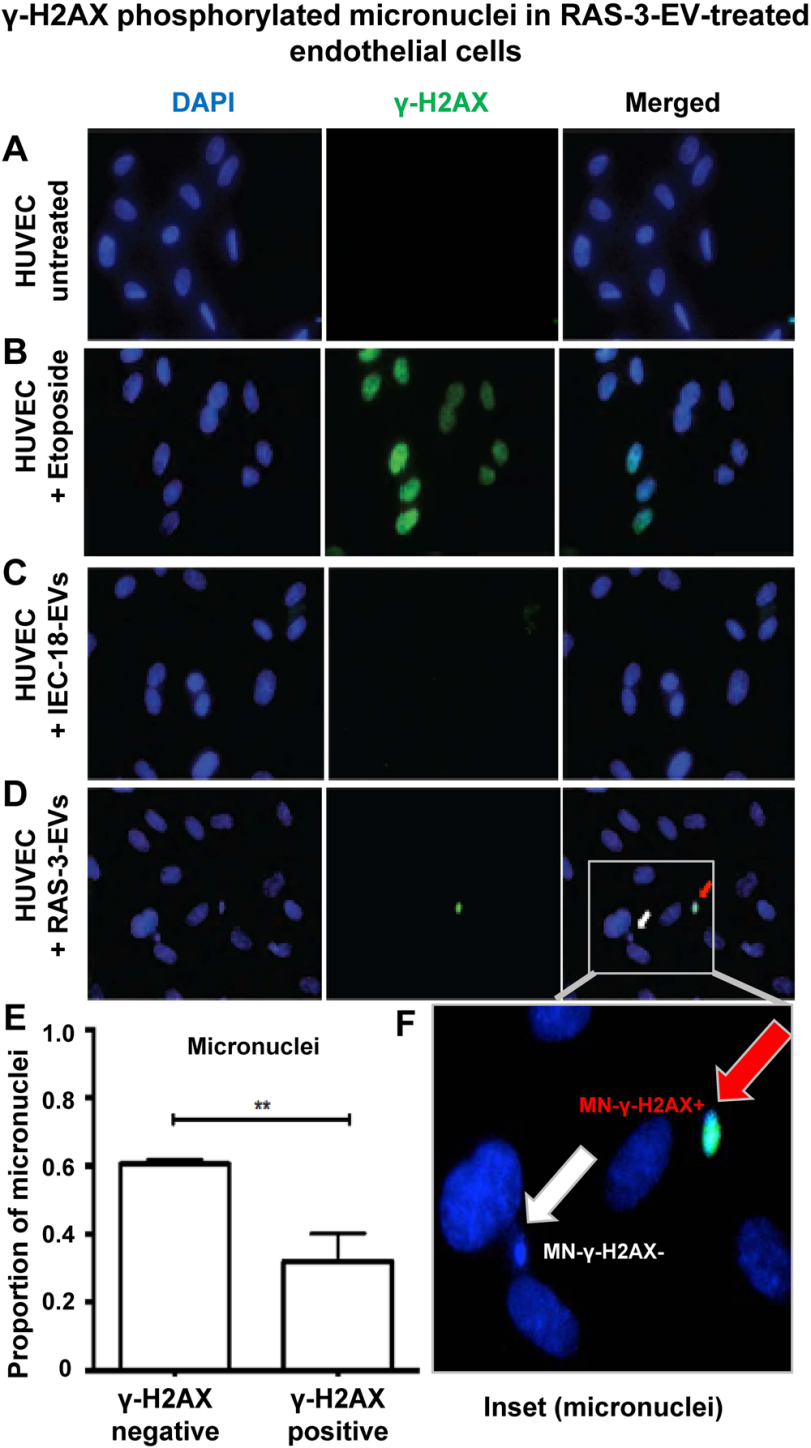
Uptake of EVs from RAS-driven cancer cells triggers features of genetic instability in endothelial cells. (**A)** Untreated HUVEC. (**B)** HUVEC treated with etoposide. (**C)** HUVEC treated with IEC-18 EVs. (**D)** HUVEC treated with RAS-3 EVs (DAPI – blue; anti-phospho-histone γH2AX – green). (**E)** Quantification of endothelial micronuclei with and without γH2AX staining. (**F)** Endothelial micronuclei with and without γH2AX staining (high magnification – 1,000×); **p < 0.01.

**Figure 7:**
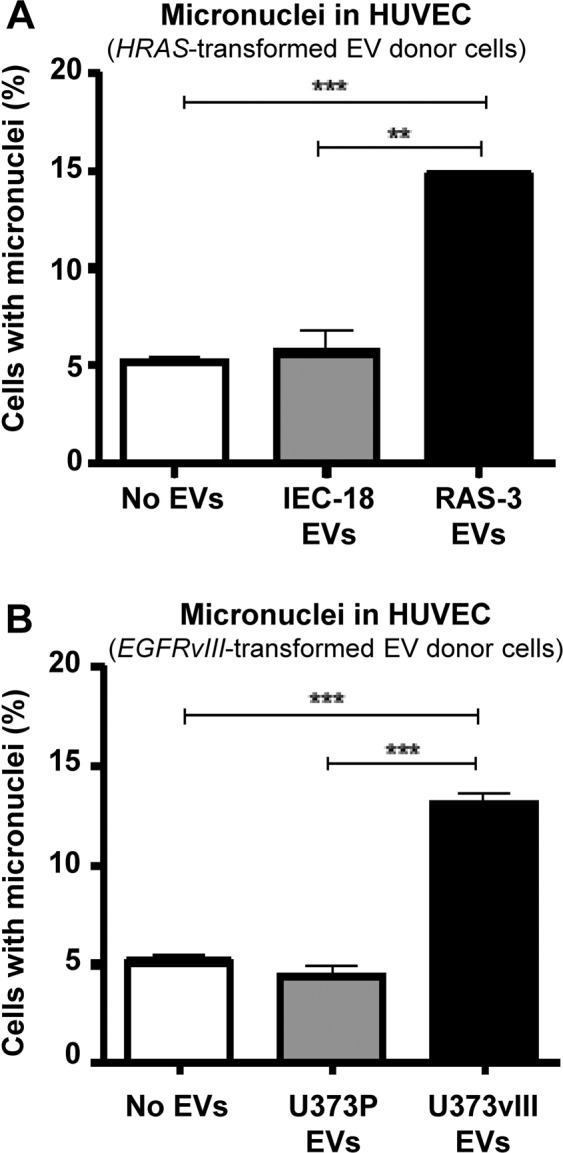
Induction of micronuclei in endothelial cells exposed to EVs from oncogene-driven cancer cells. HUVEC cells were exposed for 7 days to EVs from cancer cells expressing mutant HRAS (carcinoma) or EGFRvIII (glioblastoma). (**A)** HUVEC micronuclei count upon treatment with control, IEC-18- or RAS-3-derived EVs. (**B)** HUVEC micronuclei count upon treatment with control, U373P- or U373vIII-derived EVs; **p < 0.01; ***p < 0.0001.

To extend the aforementioned observations we also tested EV-treated endothelial cells for markers of DNA damage response such as phosphorylation of histone γH2AX (Fig. 6). Interestingly, we did observe this signal in HUVEC exposed to RAS-3 EVs, but not in those treated with IEC-18 EVs. While this signal was relatively weak and spotty in comparison to the positive control (cells treated with etoposide), it was interesting to note that phosphorylated γH2AX uncharacteristically localized to perinuclear areas rather than to nuclei themselves. Upon staining with DAPI, these spots were found to colocalize with a subset of micronuclei (Fig. 6A–F). To assess the extent of this micronuclei formation process, HUVEC cultures treated with EVs from RAS-3 or IEC-18 cells were stained with DAPI and analyzed by fluorescent microscopy. As shown in Fig. 7A, the exposure to RAS-3 EVs raised the fraction of micronuclei containing cells from approximately 5% to almost 15%, while EVs from IEC-18 cells did not exert any measurable influence. Similar assays were also conducted with HUVEC treated with EVs from indolent glioma cell line (U373P) and their aggressive variant expressing oncogenic EGFRvIII (U373vIII)^31^. Once again, endothelial cells exhibited a considerable increase in micronuclei formation in the presence of EVs from U373vIII cells, but exposure to U373P EVs was inconsequential (Fig. 7B).

We also observed proliferative responses (Supplementary Fig. S6) and micronuclei formation by HUVEC cells incubated with EVs isolated from patient-derived human glioma stem cells (GSCs; Supplementary Fig. S7). These cells exhibit distinct molecular characteristics defined as either a proneural (PN) or mesenchymal (MES) phenotype, each of which can be further modified by serum-induced differentiation, resulting in dramatic changes in vesiculation profiles^32^. While EVs from MES GSCs (GSC83) stimulated HUVEC micronuclei formation regardless of differentiation, this effect was somewhat diminished in the case of differentiated PN GSCs (DIFF-PN-GSC; GSC157 line; Supplementary Figs. S7A,B). This is of interest as differentiated GSC157 cells were earlier found to change their EV emission profile and proteome^32^. Of note GSC83 cells carry oncogenic EGFRvIII mutation while GSC157 are EGFR-negative^32,33^.

### Abrogation of EV-induced micronuclei formation in endothelial cells with disrupted tumor suppressor pathways

Since RAS-3 EV-induced micronuclei formation was paralleled by the upregulation of TP53 we examined this effect in two different immortalized human endothelial cell lines of either cerebral (HBEC-5i) or cutaneous (HMEC-1) origin, in which TP53 and Rb pathways were inactivated through the expression of SV40 LT antigen^34^ (Supplementary Fig. S8A,B). Notably, both cell lines exhibited somewhat higher background levels of micronuclei (compared to HUVEC), which remained relatively unchanged in the presence of RAS-3 EVs. Finally, the effects of RAS-3 EVs on micronuclei formation were not restricted to endothelial cells, but also occurred in normal human astrocytes (NHAs) similarly exposed to these EVs (Supplementary Figs. S9, S10). Collectively, these results suggest that, in addition to other regulatory activities attributed to cancer EVs^35^, their effect may also lead to chromatin perturbations in normal host cells including angiogenic endothelium.

### Distinct protein cargo of cancer EVs capable of inducing micronuclei formation in recipient cells

We reasoned that while transfer of oncogenic DNA is a striking feature associated with chromatin perturbations in endothelial cells exposed to cancer EVs other molecular differences may also be involved. To this effect we analyzed the proteome datasets of EVs from oncogene-driven cancer cell lines (RAS-3, U373vIII and GSC83) capable of paracrine induction of endothelial micronuclei^32,36^. Interestingly, this survey revealed the presence of 19 common hits in these EVs, including proteins involved in calcium binding (ANXA1, ANXA2, PDCD6), stem cell phenotype (CD44) and genetic instability (CEP55)^32,37,38^. In contrast, EVs from corresponding cells with no micronuclei inducing capability (IEC-18, U373P) and those that were variable in this regard (e.g. upon differentiation, Supplementary Fig. S7) contained no EGFRvIII and expressed less aggressive phenotype (GSC157) contained vastly divergent cargo and shared only 2 common proteins (VIME, PXDN) (Supplementary Figs. S11, S12). While the mechanistic role of these and other constituents of the EV cargo remains to be studied, their differential expression patterns are in line with distinct biological activities of EVs from aggressive versus indolent cellular populations.

## Discussion

Our study explores the role of EVs in intercellular propagation of genomic instability triggered by oncogenic transformation. We observed that the expression of oncogenic HRAS in cancer cells is associated with large scale aberrations in the chromatin architecture, including formation of micronuclei with non-random content of specific chromosomes. In these cells, changes in nuclear envelope were accompanied by an increase in cytoplasmic chromatin and extracellular release of genomic DNA from cancer cells, primarily through formation of small EVs.

Upon contact with normal human endothelial cells the RAS-3 derived EVs underwent internalization, retention and exhibited a range of biological activities including angiogenesis-like migratory responses and hitherto unexpected formation of micronuclei reminiscent of features associated with genotoxic stress. Thus, genetically unstable oncogene-driven cancer cells (such as RAS-3) triggered features of genetic instability in normal endothelial cells in conjunction with intercellular transfer of EVs containing genomic DNA and distinct protein cargo. Such processes may, at least in some cases, contribute to cancer-specific aberrations of the vascular growth associated with tumor neovascularization^2^.

Structural aberrations in the nuclear architecture and genetic instability are among the hallmarks of malignant growth^39,40^. In this regard, mutant RAS was reported to trigger phenotypically apparent mutations within only a few of population doublings^21^. In our hands, HRAS-mediated cellular transformation was also accompanied by signs of genomic instability, including multipolar mitoses, nuclear budding, nucleoplasmic bridges, and micronuclei formation, none of which were observed in isogenic non-transformed parental cells. Surprisingly, SKY assays revealed that the vast majority of micronuclei in RAS-3 cells were derived from large chromosomes 1 (21%), 2 (25%) or their combination (23%). Earlier reports suggest that micronuclei may accompany self-replicating chromatin units, such as double minute (DM) chromosomes containing oncogenic MYC, sometimes resulting in the active extracellular release of these structures from cancer cells^25^. While such a process could contribute to the pool of extracellular genomic DNA, this scenario is unlikely in the case of RAS-3 cells used in our experiments, for several reasons. These cells harbor exogenously introduced and integrated oncogenic *HRAS* sequences (rather than DM) and, in spite of enrichment for chromosomes 1 and 2 in micronuclei, they release EVs containing DNA that is representative of the entire cellular genome^23^. This disparity in conjunction with our live microscopy observations (unpublished) suggests an extremely rare expulsion of micronuclei from RAS-3 cells.

Instead, our immuno-gold EM studies support the notion that oncogenic RAS triggers formation of extranuclear chromatin deposits possibly resulting in contact between genomic DNA and intracellular membranes that may contribute to one or more pathways of EV biogenesis^14^. An earlier study suggested that RAS may upregulate autophagy machinery, including LC3 protein, which may, in turn, compromise the integrity of the nuclear envelope by interacting with lamin B1, leading to cytoplasmic chromatin deposits ^24^. Moreover, autophagy proteins have already been independently implicated in the formation of exosomes^26^, emission of nucleic acids and their binding proteins^41^ and in the release of nucleosomes from cancer cells^27^. Our data extend this line of work by suggesting that pharmacological inhibition of autophagy, using chloroquine, reduces the release of EV-associated oncogenic DNA (but not EVs themselves) in cancer cells driven by oncogenic HRAS. Whether such treatment would impact the integrity of tumor blood vessels remains to be investigated.

Genomic DNA is detectable in EV preparations of culture supernatants produced by a wide array of viable cancer cell lines and is found in biofluids of tumor-bearing mice and cancer patients^8,23,42–46^. This includes GBM cells driven by EGFRvIII and patient-derived GSC lines, albeit with a variable extent of extracellular DNA release (our unpublished observation). While the linkage between the DNA emission process and exosome biogenesis has been questioned^27^, there is ample evidence of intercellular transfer of oncogenic DNA *via* mechanisms involving different subtypes of EV and apoptotic bodies, often profoundly impacting various target cell populations^23,47–50^. These observations gave rise to the notion that normal cells could be converted into a tumorigenic-like state (horizontal transformation) through a horizontal gene (oncogene) transfer akin to processes occurring in plants and bacteria and mediated, at least in part, by DNA-containing EVs or particles^47,48^. EVs were also implicated in similar processes involving oncogenic RNA^50,51^, or proteins^52^. While intriguing, experimental transformation based on oncogenic EV transfer between cells tends to be transient in nature^12,19,29^ and the extent of full-blown tissue-to-tissue transformation in human pathology has not been documented and deserves further scrutiny^12^.

On the other hand, EVs have been implicated in various aspects of vascular regulation and pathological angiogenesis. Their effects are often attributed to intercellular transfer or deregulation of canonical angiogenic mediators, such as vascular endothelial growth factor (VEGF)^53–55^, interaction with NOTCH^56^ or EPHB2 pathways^57^, intercellular transfer of membrane receptors^58^, including bioactive oncogenes, such as EGFR and through other effects^19,20^. These processes may also involve EV-mediated delivery of coding^30^ or non-coding RNA to stromal and vascular cell compartments^59–61^. In cancer, the vascular, prothrombotic or angiogenic effects of oncogenic EVs may rely on their interactions with indolent or non-transformed cells resulting in deregulation of their natural angiogenic or coagulant potential^8,19,58^. Alternatively, cancer EVs may directly transfer oncogenic activity to endothelial cells resulting in their re-programming^20^. In the present study, we describe the latter type of interaction whereby EV-mediated transfer of oncogenic *HRAS* genomic sequences (along with other cargo) into endothelial cells coincided with pronounced biological responses including cell migration, proliferation^29^ and aberrations in the chromatin architecture.

Our observations challenge the widely held view that, unlike their adjacent cancer cells, tumor-associated endothelial cells remain genetically stable, even if they are functionally abnormal or overstimulated^1^. Instead, we propose that vascular mediators operative in cancer may, at least to some extent, be tumor-specific (as in the case of oncogenic EVs) and produce tumor-specific responses. While this question is infrequently studied, aneuploidy has been reported in mouse endothelial cells associated with experimental tumors analysed by Klagsbrun and his colleagues^62–64^. Moreover, oncogenic mutations have been detected amidst endothelial lining of glioblastoma tumor masses, an observation largely attributed to cancer stem cell trans-differentiation into endothelial-like cells^65^. Similar observations were also reported in hematopoietic malignancies where clonal chromosomal rearrangements present in cancer cells were found to occur in cells with an apparent endothelial phenotype^66,67^. Although some of these genetic events may simply signify, as suggested, the extent of cellular trans-differentiation (vasculogenic mimicry) of cancer cells or stem cells^2^, our study suggests that cancer cells may also influence the intrinsic genomic integrity of bona fide endothelial cells by transmission of EV-mediated signals. While for technical reasons our study did not explore full karyotypes of EV-treated endothelial cells future studies along these lines would be of great interest, including a detailed analysis of endothelial cell genome and epigenome, as well as their functional alterations such as responses to genotoxic anticancer therapies and radiation.

In our hands, the extent or micronuclei formation in EV-treated endothelial cells was influenced by overt oncogenic transformation of EV donor cells, such as the presence of either HRAS or EGFRvIII mutations. These events were accompanied by low levels of γH2AX phosphorylation and upregulation of TP53 expression in EV recipient cells, elements reminiscent of DNA damage response and cellular stress^68,69^. Tumor suppressors seem to play a role in these responses as immortalized endothelial cells with inactivated Rb/TP53 pathways were resistant to the micronuclei-inducing effects of cancer cell EVs.

Whether EV effects on endothelial cells can be attributed to intercellular transmission of genomic DNA^29,70^, ectopic activity of oncogenic signaling modules^20^, or more complex effects of other EV cargo is presently unclear. Of interest is the observation that among three different aggressive cancer cell populations that produce micronuclei-inducing EVs (RAS-3, U373vIII and GSC83) the proteomes of these vesicles contain 19 common proteins, including molecules already implicated in genetic instability (CEP55)^37^ or calcium binding (ANXA1, ANXA2, PDCD6). Intriguingly, calcium regulation has already been linked to genetic instability and micronuclei formation under the influence of cellular oxidants^71,72^. In contrast, corresponding proteomes of EVs from cells with no, low or variable micronuclei inducing capabilities were relatively divergent and devoid of the aforementioned common protein repertoire. Further studies are warranted to assess the functional consequences (if any) of these thought provoking differences.

We also observed that astrocytes responded to oncogenic EVs by an increase in micronuclei formation. This may suggest that multiple stromal cell populations in cancer may be vulnerable to EV-mediated genomic insults, that extend beyond effects of canonical stimulatory pathways of cell-cell communication in normal tissues^73^. For example, we did not observe micronuclei formation in endothelial cells treated with VEGF.

Overall, our study suggests that cancer-associated vascular anomalies may be induced by cancer-specific influences (qualitatively different than canonical angiogenic pathways) including EV-mediated transfer of oncogene-dependent mediators, such as extracellular mutant DNA, protein assemblies and possibly other factors. Further studies are required to examine whether these effects are found in human cancers, and whether they are responsible for vascular pathologies or represent viable therapeutic targets.

## Materials and methods

### Cells and culture conditions

U373P (Glioblastoma parental cell line), U373VIII (Glioblastoma cell line expressing the EGFRvIII oncogene), and NHA (non-tumorigenic, normal human astrocytes from Dr. Guha, UHN) were cultured in Dulbecco’s Modified Eagle’s Medium (DMEM) supplemented with 10% fetal bovine serum (FBS) (Multicell Wisent Inc, Catalog No. 085150) and 1% penicillin and streptomycin (P/S). Intestinal epithelial cell line (IEC-18) was transfected with plasmid containing human V12 mutant active c-HRAS human oncogene to produce RAS-3 cell line, as earlier described by Filmus et al (University of Toronto). IEC-18 and RAS-3 cell lines were maintained in Minimum Essential Medium Alpha Medium (AMEM) supplemented with 5% FBS, 20 mM glucose, 4mM L-glutamine (GIBCO, Catalog No. 25030-081), 10 μg/ml insulin (Sigma Chemical Co., St. Louis, MO). In addition, conditioned medium containing EV-depleted FBS was used to culture donor cell lines for EV isolation. Glioma stem cell (GSC) lines were obtained from the laboratory of Dr. Ichiro Nakano (University of Alabama, Birmingham). The two cell lines with either proneural (GSC157) or mesenchymal (GSC83) subtypes were acquired in the form of spheres from surgical samples of glioblastoma (GBM) patients. Both GSCs were maintained as spheres in the medium containing DMEM-F12 (GIBCO, Catalog No. 11320033) supplemented with 100 μg/ml EGF (GIBCO, Catalog No. PHG0311L), 100 μg/ml FGF (GIBCO, Catalog No. PHG0261), 0.2% Heparin (STEMCELL, Catalog No. 07980), 1X B27 serum free supplement (GIBCO, Catalog No. 17504044), 1% Glutamax (GIBCO, Catalog No. 35050061) and 1% penicillin-streptomycin (P/S) (GIBCO, Catalog No. 15070063). GSCs were differentiated using DMEM-F12 medium supplemented with 10% FBS, 1% P/S and 1% Glutamax. HBEC-5i, an immortalized human brain endothelial cell line (ATCC, Catalog No. CRL-3245) was cultured on 0.1% gelatin coated plates. Cells were maintained in DMEM-F12 medium supplemented with 10% FBS, 1% P/S and 40 μg/mL endothelial growth supplement (ECGS) (Sigma, Catalog No. E2759). HMEC-1, an immortalized dermal microvascular endothelial cell line (ATCC, Catalog No. CRL-3243) was maintained in MCDB131 basal medium supplemented with 10 ng/ml EGF (GIBCO, Catalog No. PHG0311L), 1 µg/ml hydrocortisone (Sigma, Catalog No. H6909), 10 mM glutamine and 10% FBS. HUVEC are normal human primary umbilical vein endothelial cells (ATCC, Catalog No. PCS-100-010) that were cultured on 0.1% gelatin coated plates and maintained using EGM-2 BulletKit media (Lonza, Catalog No. CC-3162).

### EV isolation from cultured cells

EV donor cell lines were cultured in EV-depleted conditioning medium for 3 days. The supernatant containing EVs was centrifuged at 400 g for 10 minutes followed by filtration with 0.2 μm PES filter to remove cells and cell debris^29,74^. The filtrate was further subjected to ultracentrifugation at 110,000 g for 1 hour to pellet EVs. For differential centrifugation, the filtrate was first spun at 10,000 g for 30 minutes (to separate ectosomes or P2/P3 fraction) and then at 110,000 g for 1 hour to isolate exosome-like EVs, or P4 fraction^23^). EV-DNA was isolated by proteinase K-treated lysis buffer.

### Differential isolation of EVs by filtration

Conditioned medium from EV donor cells were passed through series of filters (3 μm, 1 μm and 0.2 μm). Filters were rinsed to collect vesicular material. The final flow through was ultracentrifuged at 110,000 g for 1 hour to collect residual EVs. DNA was extracted from vesicles collected on filters as well as from EV pellet and final flow through filtrate and droplet digital PCR (ddPCR) was performed as described earlier^8^.

### Transmission electron microscopy (TEM)

IEC-18 and RAS-3 cell pellets were washed twice with wash buffer (0.1 M sodium cacodylate buffer pH 7.4) and fixed with fixative solution (2.5% glutaraldehyde in 0.1 M sodium cacodylate buffer). After 1 hour of fixing, the cells were pelleted and stored at 4 °C. The pellets were then sectioned and imaged according to standard protocols and images were captured using Tecnai 12 BioTwin 120 kV TEM^23^ by our McGill colleagues Jeannie Mui and Line Mongeon at the Facility for Electron Microscopy Research (FEMR) unit.

### Immunogold labeling

Cells were fixed with 4% paraformaldehyde and 0.5% glutaraldehyde. The fixed cells were processed for LRWhite embedding. LRWhite embedded blocks were cut into ultrathin sections (100 nm) using diamond knives. The sections were stained with primary antibody (H3 total Histones, abcam ab24834) and dsDNA antibody (Santa Cruz Biotechnology sc58749) or BrdU antibody, (Abcam ab8152), followed by gold-conjugated secondary antibody (10 nm and 20 nm). Images were taken using the Tecnai 12 BioTwin 120 kV Transmission Electron Microscope.

### Fixation and staining of cells on slides

Cells (9 × 10^5^) were cultured on autoclaved slides and incubated with 4 × 10^9^ indicated EV preparations for 7 days (or as indicated). Slides were washed with PBS and fixed with 3.7% formaldehyde/PBS for 10 minutes at room temperature (RT) in a coplin jar. Slides were then washed again 3x with PBS for 5 minutes each at RT. Following this step, slides were serially treated with 70%, 90% and 100% dilutions of ethanol for 3 minutes each at RT and subsequently air dried. Antifade DAPI (Invitrogen, Catalog No. P36935) was added on the cover slips and placed on the slides before analyzing micronuclei formation.

### Spectral karyotyping (SKY) analysis

SKY experiments were performed with SkyPaint DNA Kit from Applied Spectral Imaging (Carlsbad, CA, USA) for Rat Chromosomes and using the supplier’s hybridization protocols. We used Spectra CubeTM on a Carl Zeiss Axioplan 2 microscope and the imaging was captured using 63x oil objective. The analysis was performed using SKYVIEW 1.6.2 and 2.0 softwares. Three independent SKY assays were performed for each of the experiments.

### Fluorescence *In Situ* Hybridization (FISH)

The FISH protocol was performed according to the Metasystem guidelines. FISH assay for rat chromosomes 1 (probe XRP1 green; 405 nm), (MetaSystems, Catalog No. D-1501-FI) and 2 (probe XRP2 orange; 488 nm), (MetaSystems, Catalog No. D-1502-050-OR) was carried out at the metaphase stage, where cells were pre-treated with 10 ml of hypotonic solution (75 mM KCl)^75^. Briefly, cells were fixed with methanol and acetic acid (3:1). Fixed cells were spotted on glass slides and spread uniformly and the slides were dried. Using the ThermoBrite system from Abbott Molecular, the sample and probe were denatured at 75 °C for 2 minutes and the temperature was then lowered to 37 °C to allow the probe to hybridize for 48 hours. The slides were counter stained with anti-fade DAPI (MetaSystems, Catalog No. CD-0902-500-DA) and covered with cover slips. Images were captured using confocal microscope (Zeiss LSM780 laser scanning confocal microscope) with 63x objective (Zeiss Plan-Apochromat) and fluorescence at absorption of 552 nm and emission of 576 nm (34 channel spectral R/FL detectors).

### Interference with autophagy using chloroquine treatment

IEC-18 and RAS-3 cells were treated with different concentrations (25 μM, 50 μM, 100 μM) of chloroquine (Cell Signaling Technology, # 14774) to inhibit the autophagy process at different time points (4, 6, 8, 10, 14, 16 hours). At 50 μM concentration for 16 hours we found an optimal effect without causing overt toxicity to cells. To visualize the chloroquine effect, 50 nM of LysoTracker Green DND-26 (Thermo Fisher Scientific, Catalog No. L7526) was added to the growth medium containing drug treated cells and incubated at 37 °C for 30 minutes. Live Images were taken using confocal microscope at magnification of 400×. EVs were collected from conditioned media of both chloroquine-treated and untreated cells and DNA was isolated, quantified and assayed as indicated.

### Droplet digital PCR (ddPCR)

ddPCR assay was performed according to the manufacturer’s protocol. Briefly, each reaction consisted of ~10 ng of the template DNA, 1x ddPCR Eva Green Supermix, 1 μM forward and reverse primers in a final volume of 20 μl. For each reaction, 60 μl of Droplet Generation Oil (Bio-Rad) was applied, loaded onto cartridge and droplets were generated using the QX100 Droplet Generator (Bio-Rad). The droplets were transferred to a 96-well plate, sealed and HRAS PCR was performed with the following conditions: 1× 95 °C for 5 min, 45× (95 °C for 30 sec, 64 °C for 60 sec and 72 °C for 30 sec), and 1× 90 °C (5 min). After the PCR reaction was completed, the plate was transferred and read in QX100 Droplet Reader (Bio-Rad) and data were analyzed with QuantaSoft droplet reader software (Bio-Rad)^8^.

### Scoring of micronuclei

Genetic instability was scored by manually counting of DAPI stained cells for individual micronuclei and nucleoplasmic bridges (NPBs). About 200-300 cells were counted for each experiment and experiments were repeated at least 3 times. Criteria for micronuclei scoring were as follows: a) presence of micronuclei outside of the main nucleus b) diameter of micronuclei less than one-third of the nucleus c) the intensity of DAPI stained micronuclei similar to that of the main nucleus d) micronuclei in the same focal plane as the nuclei.

### MTS cell proliferation assay

HUVECs were seeded in 96 well plates at a density of 7 × 10^3^ cells/well with complete media for 24 hr. The following day the cells were washed and treated with EVs (30ug (protein)/mL) isolated from PN GSCs (GSC157) or MES GSCs (GSC83) in DMEM containing 1%FBS. Cell proliferation was then assessed, at different time intervals (1,3 and 6 days), by the colorimetric MTS reduction method (Promega, Catalog No. 43580, CellTiter96 Aqueous One Solution Cell Proliferation Assay - MTS) following the manufacturer’s instructions. The absorbance was measured at 490 nm using a microplate reader.

### EV proteomics and statistical analysis

Equivalent protein amounts from EV preparations were loaded onto 10% SDS-PAGE pre-cast gel (BioRad) followed by trypsin digestion of proteins under reducing conditions within the stacking gel as previously described^76^. The resulting lyophilized peptides were solubilized and loaded onto Thermo Acclaim Pepmap (Thermo, 75 μM ID X 2 cm C18 3 μM beads) pre-column and then onto an Acclaim Pepmap Easyspray (Thermo, 75 μM X 15 cm with 2 μM C18 beads) analytical column separation using a Dionex Ultimate 3000 uHPLC at 220 nl/min as described in^36^. Peptides were sequenced by Thermo Orbitrap Fusion mass spectrometer and data was analyzed using Scaffold Q+ software (version 4.8.4) as described earlier^36^. Relative data abundance of all the EV proteomes were quantified using total ion chromatogram (TIC) and p values were calculated using student’s t test by Scaffold Q+ software with significance threshold of 0.05^36^. In addition, Heatmaps of EV proteomic data were generated using MultiExperiment Viewer (MeV) version 4.9.

### Transwell migration assay

Gelatin coated 8.0 μm transwell inserts (ThermoFisher Scientific, Catalog No. 353097) were placed in 24-well plates and HUVEC (2 × 10^3^) cells were plated into the inserts on day 0 of the experiment. IEC-18 EVs, RAS-3 EVs, or no EVs (negative control) were added on to the HUVEC cells to stimulate their migration. After an incubation of seven days, the inserts containing cells were fixed with 3.7% formaldehyde for 5 minutes and washed 3x with PBS for 5 minutes each time. This step was followed by staining with 0.5% crystal violet solution (0.5 g crystal violet, 20 ml methanol, 80 ml water; mixed and filtered with 0.45 μm filter) for 10 minutes and washed with PBS until no excess stain was left on the inserts. Finally, the non-migrated cells (inside the inserts) were removed manually by gently swabbing the inside surface of each insert using cotton swabs without damaging the insert membrane. Finally, the inserts were examined under the light microscope to evaluate the number of cells that migrated through the membrane to the bottom surface of the insert.

### Immunofluorescent staining

The DNA damage and repair sustained by EV recipient cells was measured using phosphorylation of H2AX at serine 139 position (γ-H2AX). This signal was detected using OxiSelect DNA Double Strand Break (DSB) staining kit (Cell Biolabs, Inc, Catalog No STA-321). HUVECs (4.5 × 10^3^) were cultured in 96-well plate and treated with indicated EV concentrations. Cells were fixed with 3.7% formaldehyde/PBS for 10 minutes at room temperature (RT) and washed 2x with PBS for 5 minutes each, treated with ice-cold 90% methanol and incubated for 10 minutes at 4 °C. Following this step, the cells were washed with PBS again and incubated with a blocking buffer (1% BSA/PBS) for 30 minutes at RT on an orbital shaker. The liquid was aspirated and cells were incubated with anti-phospho-histone antibody solution (1:100 in 1% BSA/PBS) for 1 hour at RT on an orbital shaker. Wells were then washed again with PBS-T (5 times) and incubated with FITC conjugated secondary antibody (1:100 in 1% BSA/PBS) for 1 hour at RT on an orbital shaker. Following this step the cells were washed 5x with PBS-T, liquid aspirated and 200 μL 1X PBS was added with DAPI to each well. Images were captured using fluorescent microscope. As an additional assay to measure DNA damage response, HUVECs were treated with indicated EVs and prepared for immunocytochemistry (ICC), as described above. The cells were subsequently incubated with anti-P53 antibody solution (1:100 in 1% BSA/PBS) for 1 hour and washed 5x with PBS. Following the incubation with Alexa fluor 594 conjugated secondary antibody (1:100 in 1% BSA/PBS) for 1 hour at RT and 5x with PBS for 5 minutes each. Cells were then layered with 200 μL of 1X PBS with antifade DAPI before being visualized under fluorescence microscope.

### Data collection, interpretation and statistical analysis

Several independent experimental replicates (as indicated) were collected expressed as mean or medium of individual measurements +/− standard deviation (SD) and statistically analyzed using two-tailed t-test and ANOVA with the p-value threshold of 0.05.

## Supplementary information

Supplemental information.
