# Supplementary material for: Extracellular vesicles from genetically unstable, oncogene-driven cancer cells trigger micronuclei formation in endothelial cells
Source: Sci Rep. 2020 May 22;10:8532. doi: 10.1038/s41598-020-65640-7 (PMC7244541; doi:10.1038/s41598-020-65640-7)
Supplement: Supplementary file 1 — Supplemental information. [file 41598_2020_65640_MOESM1_ESM.pdf]

Extracellular vesicles from genetically unstable, oncogene-driven cancer cells trigger micronuclei formation in endothelial cells

Shilpa Chennakrishnaiah[a], Thupten Tsering[a], Caroline Gregory[a], Nadim Tawil[a], Cristiana Spinelli[a], Laura Montermini[a], Nicoloas Karatzas[a], Saro Aprikian[a], Dongsic Choi[a], Ludger Klewes[b], Sabine Mai[b], Janusz Rak[a]

Supplementary Figures

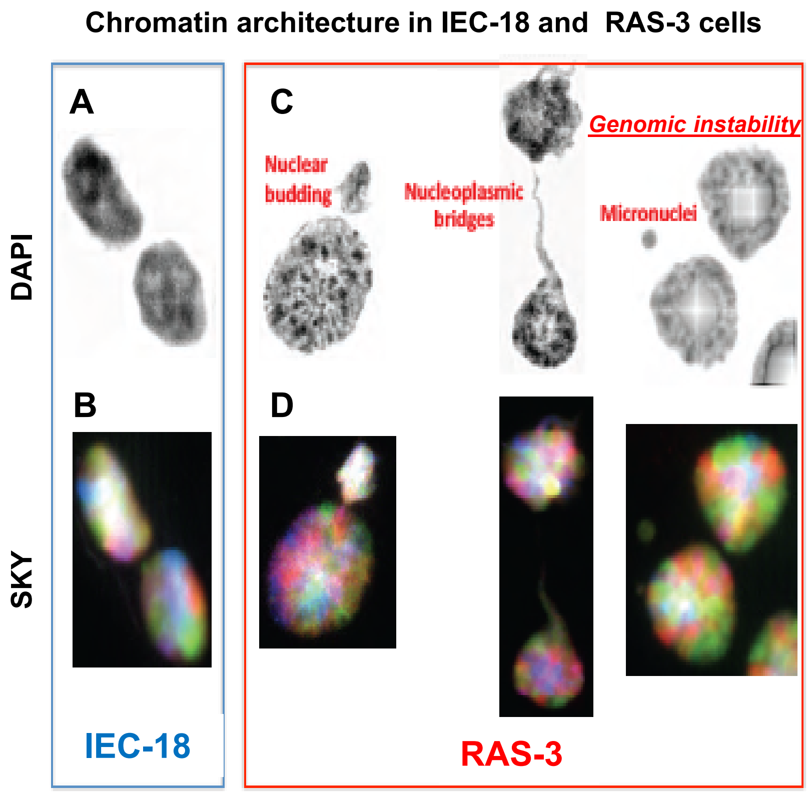

Figure S1. DAPI and SKY staining reveals alterations in the chromatin architecture of IEC-18 cells following transformation with mutant HRAS oncogene

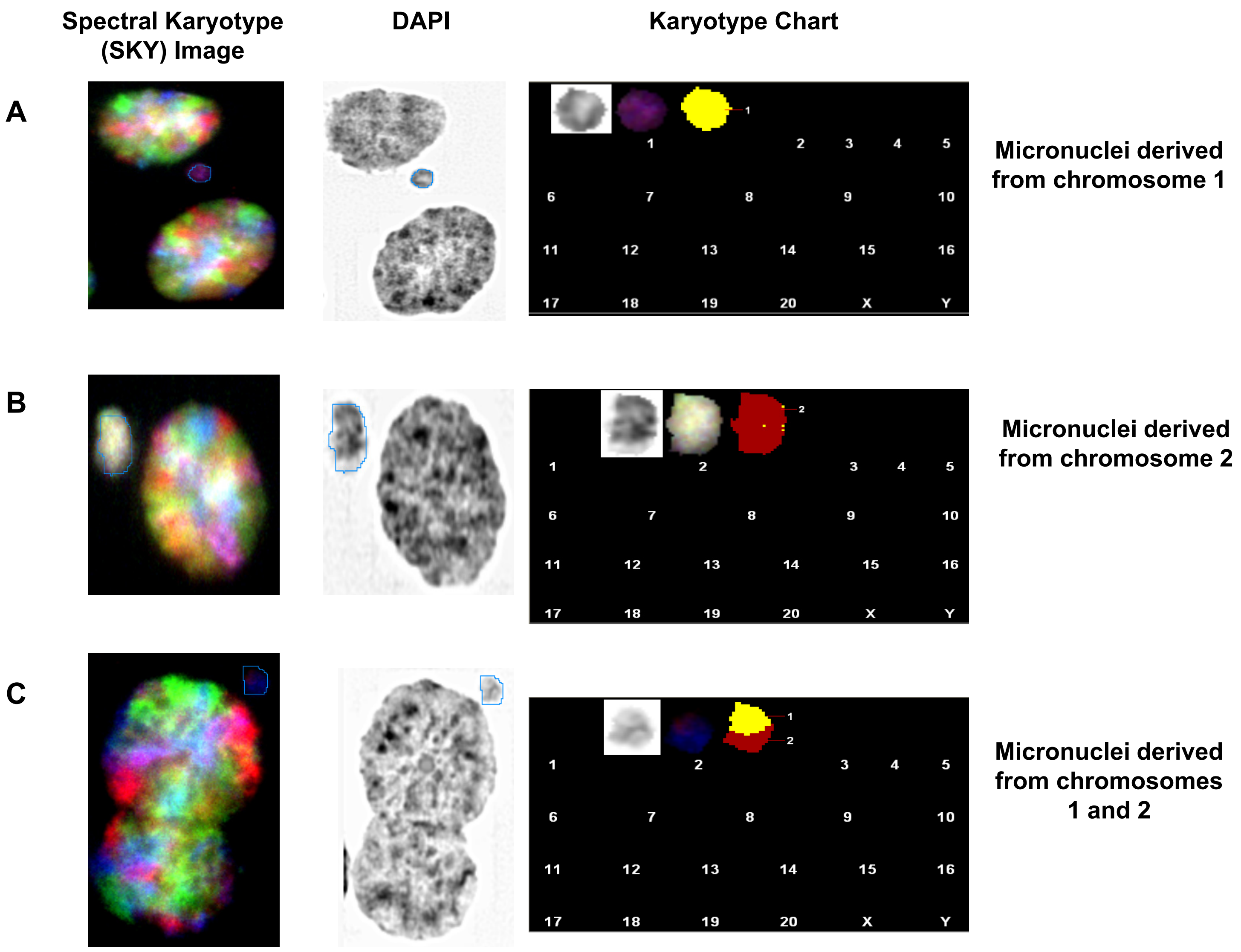

Figure S2. Analysis of chromosomal content in micronuclei of RAS-3 cells

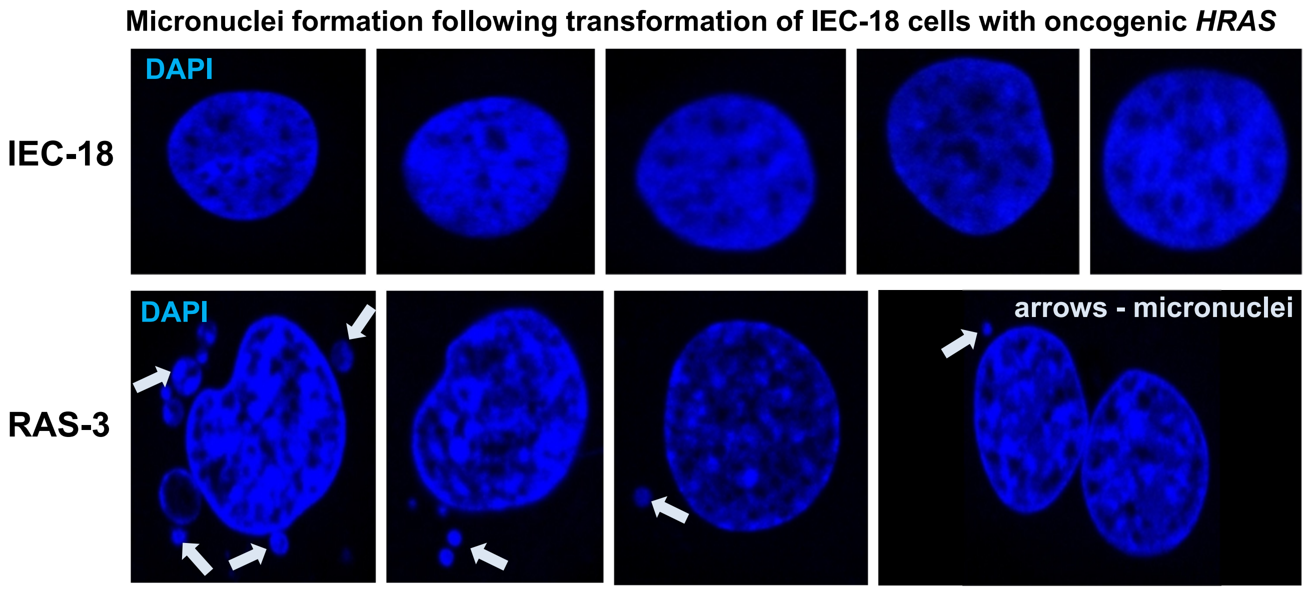

Figure S3. DAPI staining reveals the impact of oncogenic HRAS on formation of micronuclei in cultured epithelial cells

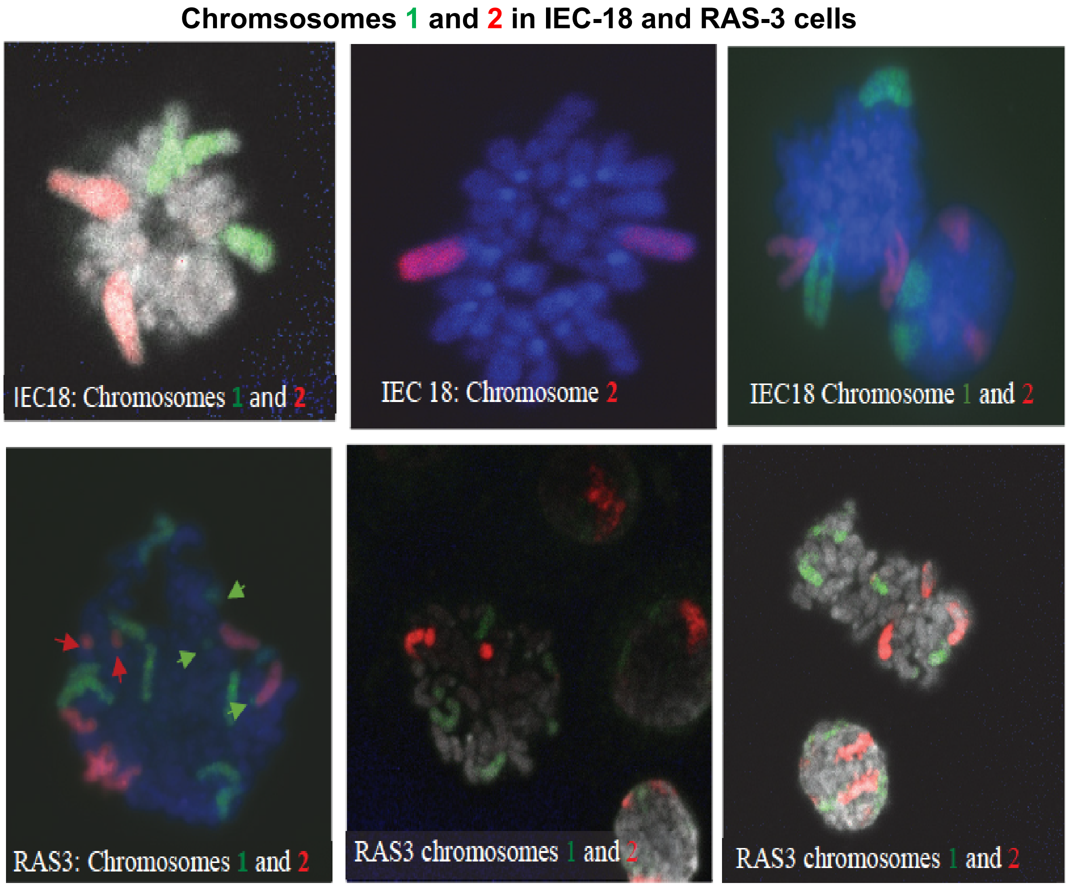

Figure S4. FISH staining reveals multiple chromosome 1 and 2 signals in nuclei of *HRAS*-transformed (RAS-3) cells

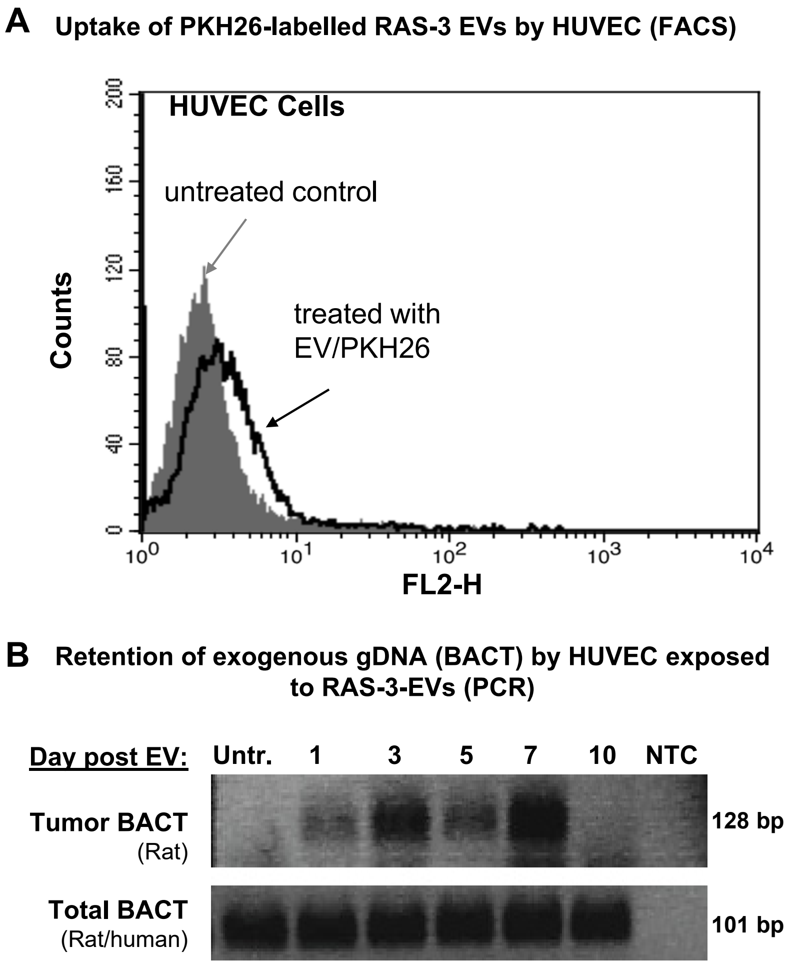

Figure S5. EV-mediated horizontal transfer of oncogenic *HRAS* DNA sequences from cancer cells to endothelial cells

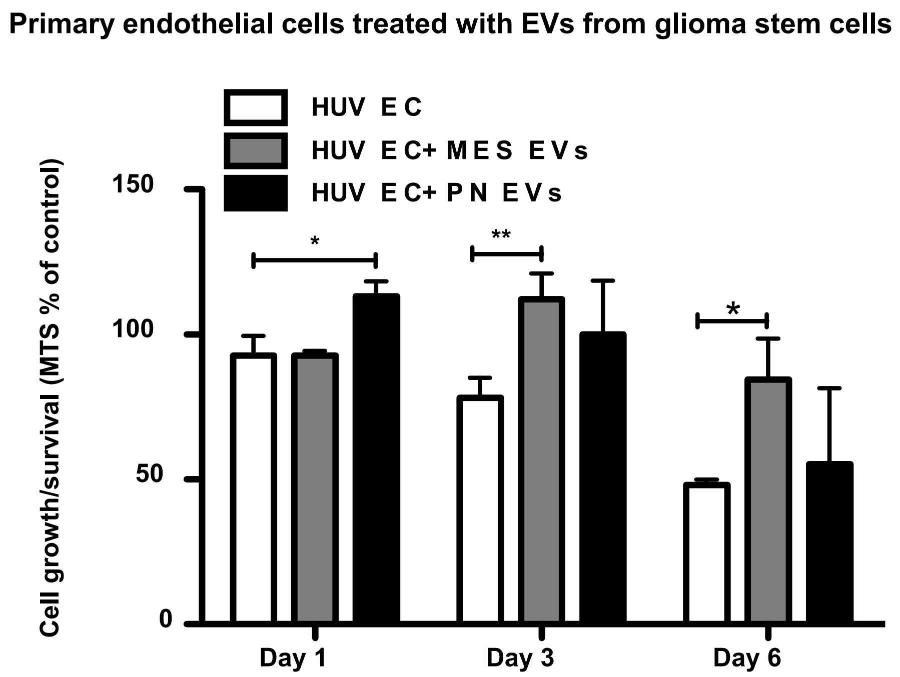

Figure S6. Stimulation of endothelial cell proliferation/survival by EVs from mesenchymal (MES) and proneural (PN) GSCs.

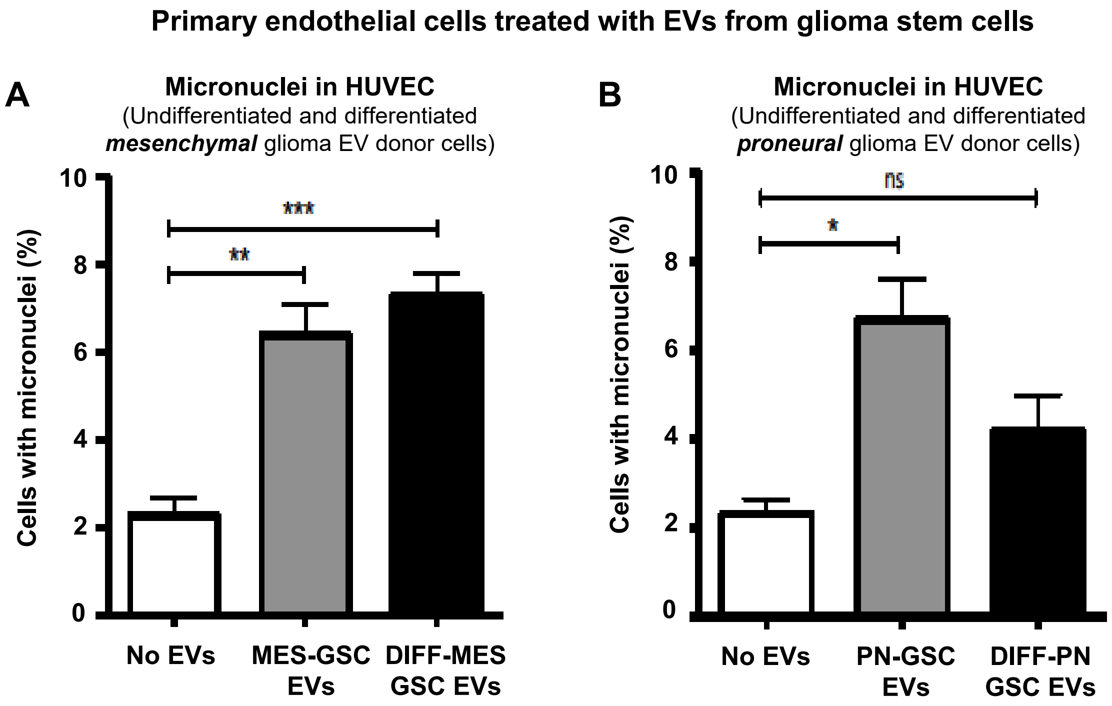

Figure S7. Differential effects of EVs from proneural and mesenchymal glioma stem cells (GSCs) and their differentiated counterparts (DIFF) on micronuclei formation by endothelial cells

Immortalized endothelial cells treated with EVs from normal and cancer cells

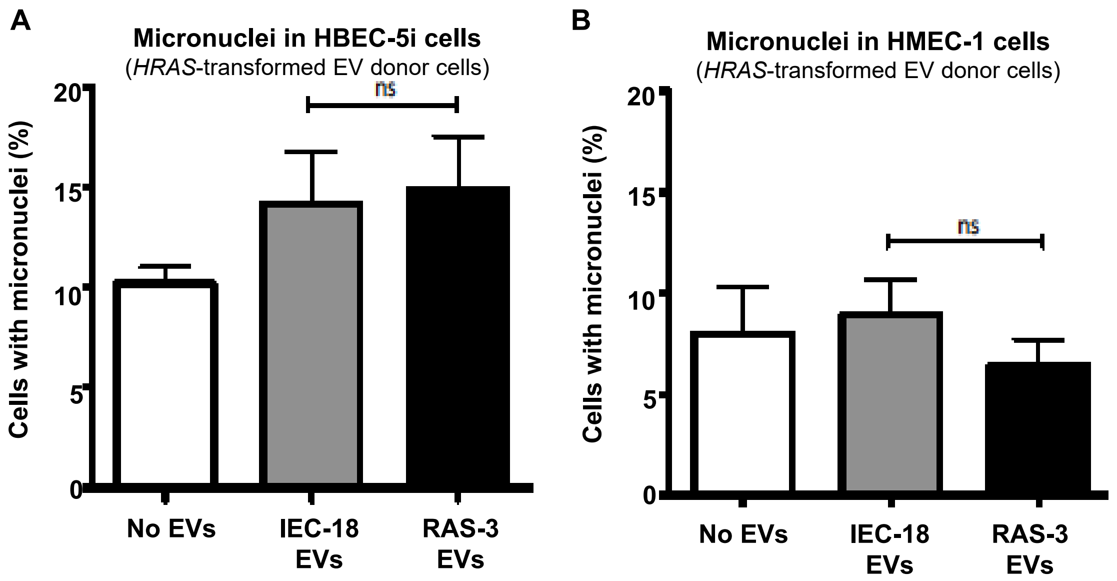

Figure S8. The effects of cancer EVs on endothelial micronuclei formation is abrogated in immortalized endothelial cell lines

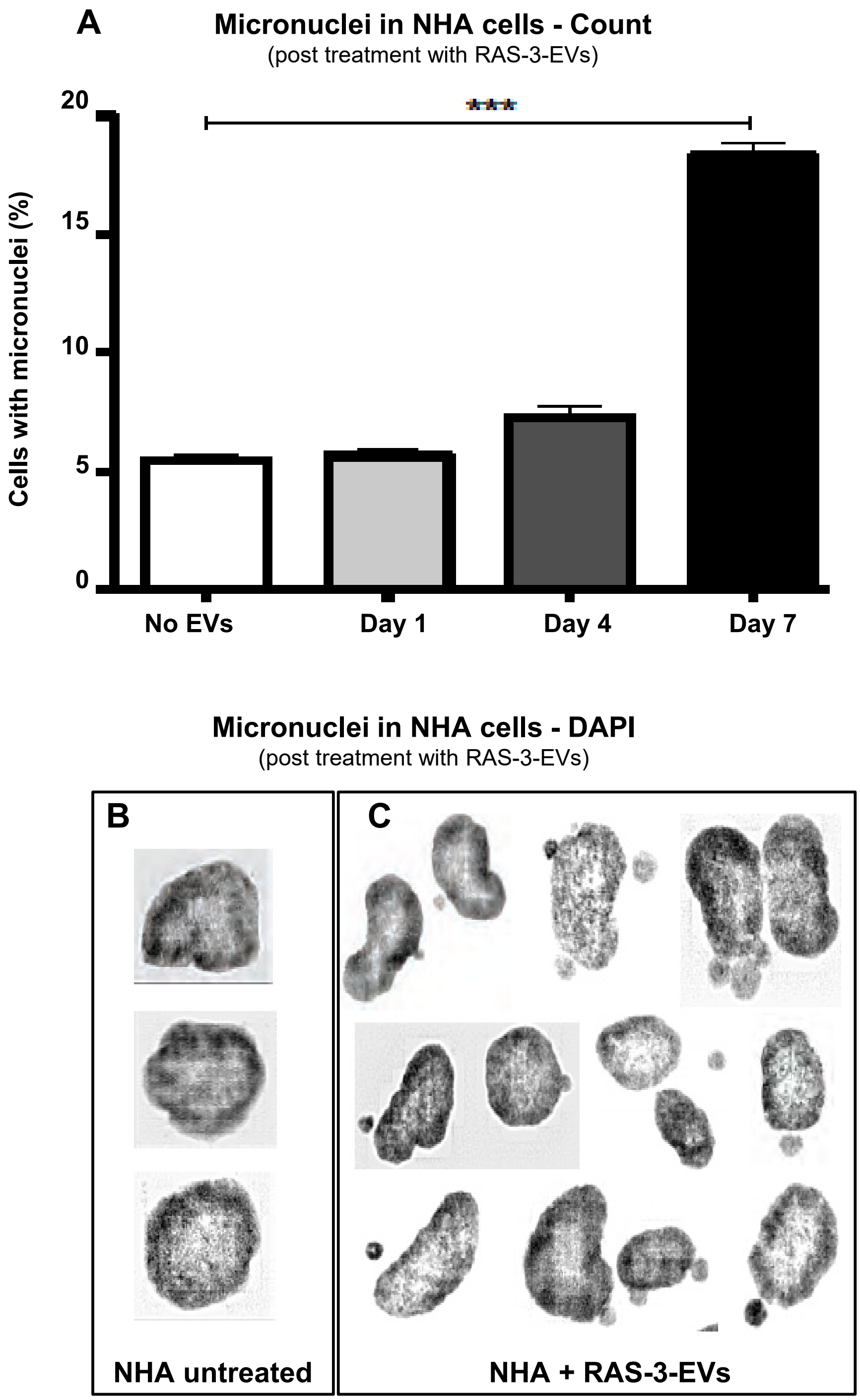

Figure S9. Formation of micronuclei in astrocytes (NHA) treated with EVs from HRAS-transformed RAS-3 cancer cells

HTERT-immortalized human astrocytes (NHA) treated with EVs from cancer cells

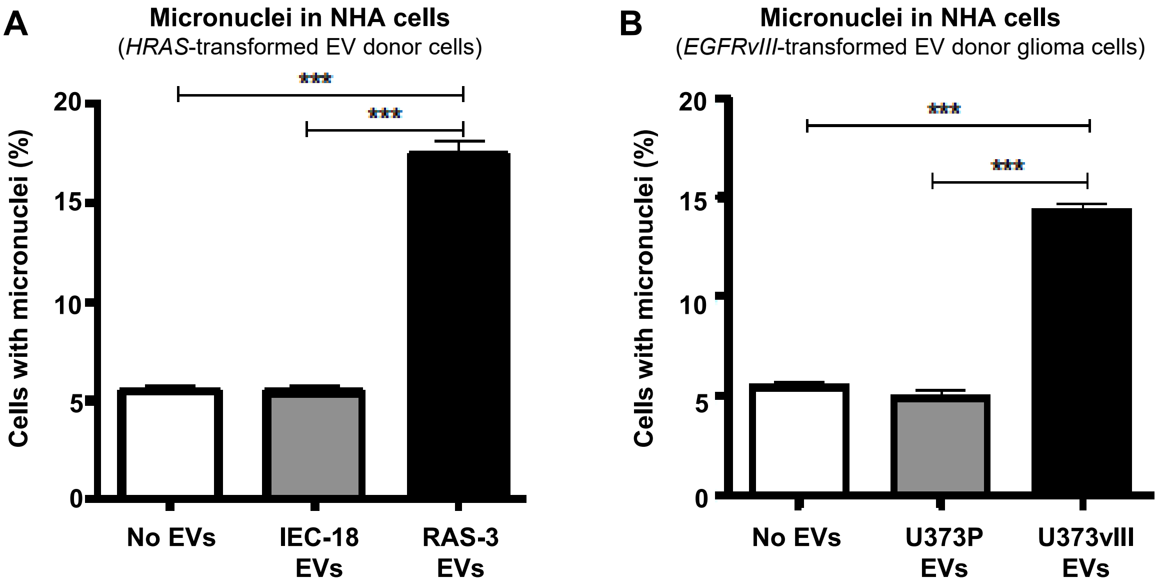

Figure S10. EVs from cells transformed with HRAS or EGFRvIII oncogenes, but not from their indolent isogenic counterparts, trigger formation of micronuclei in immortalized human astrocytes

A EV proteins shared between cancer cell lines capable of inducing endothelial micronuclei

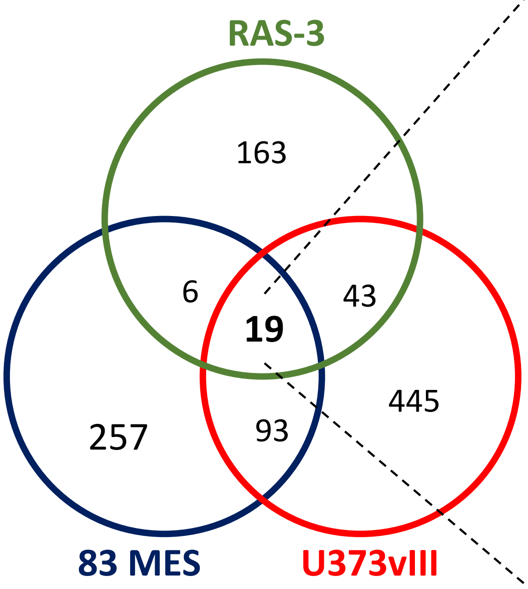

Protein (≥ 4 peptides)	Full name (19 identified)
ANXA1	Annexin A1
ANXA2	Annexin A2
AP2B1	AP-2 complex subunit beta
CD109	CD109 antigen
CD44	CD44 antigen
CD9	CD9 antigen
CDC42	Cell division control protein 42
CEP55	Centrosomal protein
DNJA1	DnaJ homolog subfamily A member 1
EHD4	EH domain-containing protein 4
EPHA2	Ephrin type-A receptor
IGSF8	Immunoglobulin superfamily member 8
LAMB1	Laminin subunit beta-1
LAMC1	Laminin subunit gamma-1
PDCD6	Programmed cell death protein 6
PPIA	Peptidyl-prolyl cis-trans isomerase A
RAB10	Ras-related protein Rab-10
RAC1	Ras-related C3 botulinum toxin substrate 1
VIME	Vimentin

B EV proteins shared between cancer cell lines not capable of inducing endothelial micronuclei

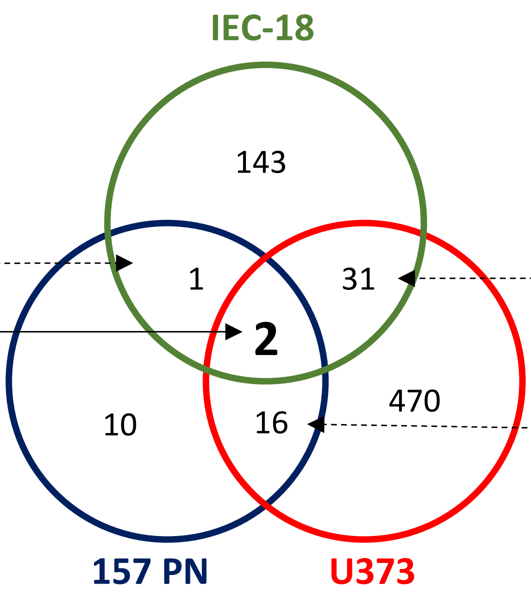

Protein
ACTB1

Proteins
PXDN
VIME

Proteins
A2MG
ALBU
CO3
FINC
GELS
K1C10
K1C14
K1C16
K1C17
K1C9
K22E
K2C1
K2C5
K2C6B
MFGM
SAHH

Proteins
ACLY
ACTN4
AHNK
ALBU
ALDOA
ANXA1
ANXA2
ANXA4
ANXA5
ANXA6
AP2A1
AP2B1
CD81
CPNE3
DNJA2
EZRI
FLNA
GNAI2
GSTP1
IGSF8
IST1
MYO1C
MYOF
PDIA3
PDC6I
PRDX1
RAB10
RAB1A
RAB2A
RAP1B
SDCB1

Figure S11. Common EV-associated proteins released from cancer cells capable of paracrine induction of endothelial micronuclei

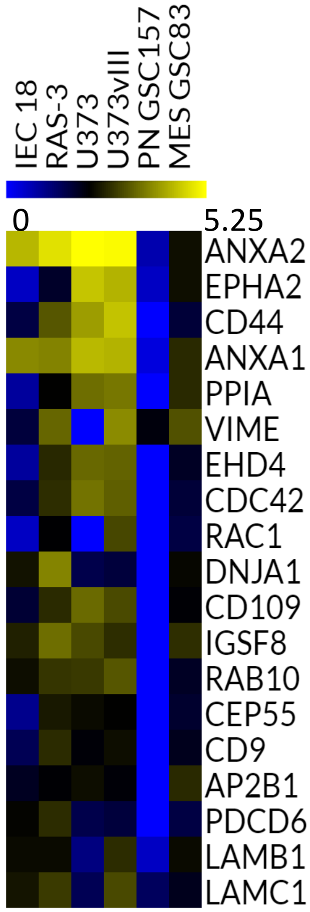

Figure S12. Comparative EV content of proteins shared between cancer cells capable of inducing micronuclei formation in endothelial cells
